# Medial closure supracondylar femoral osteotomy: an effective solution for long-term treatment of arthritic valgus knee?

**DOI:** 10.1186/s10195-021-00600-z

**Published:** 2021-09-15

**Authors:** Francesco Mattia Uboldi, Martino Travi, Daniele Tradati, Alessio Maione, Andrea Fabio Manunta, Massimo Berruto

**Affiliations:** 1 ASST Centro Specialistico Ortopedico Traumatologico Gaetano Pini–CTO, Università Degli Studi Di Milano, P.zza A. Ferrari 1, 20122 Milan, Italy; 2 Università Degli Studi Di Sassari, AOU Sassari, V.le San Pietro 43b, 07100 Sassari, Italy; 3 ASST Centro Specialistico Ortopedico Traumatologico Gaetano Pini–CTO, U.O.C. Ortopedia e Traumatologia Pediatrica, P.zza A. Ferrari 1, 20122 Milan, Italy

**Keywords:** Knee, Femur, Valgus, Osteotomy, Unicompartmental osteoarthritis

## Abstract

**Purpose:**

The aim of this work was to retrospectively analyze the clinical, subjective, and radiological results of medial closing-wedge distal femur osteotomy (MCW-DFO) for the treatment of osteoarthritis (OA) in valgus knee at medium- to long-term follow-up.

**Materials and methods:**

A total of 57 patients (62 knees) treated with MCW-DFO between 1984 and 2018 were included in the study. Patient age at the time of the surgery ranged between 28 and 61 years (average: 48 years). All patients with a minimum follow-up of 4 years were contacted to request for them to undergo clinical, subjective, and radiological evaluation. Preoperative hip–knee–ankle (HKA) angle (i.e., preoperative valgus malalignment) was 8.6° ± 2°. Patients were evaluated using the following scales: the Knee Injury and Osteoarthritis Outcome Score (KOOS), the Knee Society Score (KSS), the International Knee Documentation Committee (IKDC), the Visual Analog Scale (VAS), and the Numeric Rating Scale 11 (NRS-11).

**Results:**

Mean follow-up was 11.6 ± 4.9 years, and a total of 17 patients (20 knees) were available for the last examination. At maximum follow-up, 4 patients underwent conversion to a total knee replacement (20%); their survival rate was 100% at 10 years and 66.7% at 15 years, as estimated using the Kaplan–Meier curve. The subjective Knee Society Score improved on average from 37.7 ± 10 to 63.9 ± 15.4. The objective Knee Society Score improved on average from 42.2 ± 11.7 to 75 ± 22.5. The pain detected through the VAS and NRS-11 scales improved from 56.7 ± 12.9 to 42 ± 17.1 and from 5.8 ± 1.1 to 4.4 ± 1.7, respectively. Thirteen patients (70%) required hardware removal at an average time of 19 ± 4 months due to a local nuisance.

**Conclusions:**

MCW-DFO can improve symptoms in patients with osteoarthritis in a valgus knee at medium- to long-term follow-up, reducing the progression of osteoarthritis in properly selected patients.

## Introduction

Osteoarthritis of the knee is a frequent condition [1], with a higher occurrence in subjects affected by axis deviation of the lower limbs. In patients affected by osteoarthritis, valgus deformity occurs less frequently than varus deformity, affecting only 10–15% of their knees [2]. Unfortunately, there are multiple factors that could lead to a valgus deformity of the lower limb, and no specific recurrent cause has been identified. The most common theory is that the valgus deformity originates from the bone and might be localized mostly in the femur [3]; this hypothesis is supported by some comparative studies in which better results were obtained through femoral rather than tibial osteotomy to correct valgus knee. Recent studies only partly support this theory; as a matter of fact, this deformity was found to originate from hypoplasia of the lateral femoral condyle in most cases, although a tibial component was associated [4] in approximately half of the cases. Correct patient selection for* distal femoral osteotomy* (DFO), as well as high tibial osteotomy, is mandatory for achieving good outcomes. First of all, medical comorbidities should be addressed, as well as the patients’ functional expectations. Obesity, evaluated as 1.32 times the normal weight or a BMI greater than 30 kg/m^2^, has been associated with poorer outcomes [5]. The presence of inflammatory disorders should be addressed as well; in this population, valgus deformity is common but osteotomies are normally contraindicated [6]. Patients considered for a DFO should be less than 65 years old, active, and affected only by lateral arthritis; however, not only the patient’s age but also their activity level, lifestyle, and general health must be taken into consideration [7]. DFO should be considered in the presence of isolated lateral compartment arthritis, but the tibial axis should always be evaluated. A bifocal osteotomy should be considered in order to exert a combined action on both femur and tibia, especially for wide ranges of deviations [4]. Once it has been established that a distal femoral osteotomy is indicated, the most appropriate surgical technique should be selected. However, there is no general agreement regarding the best technique; each surgeon evaluates the advantages and disadvantages of each method. DFO options include* medial closing-wedge distal femoral osteotomy* (MCW-DFO) and *lateral opening-wedge distal femoral osteotomy* (LOW-DFO). MCW-DFO has some advantages: (i) a single osteotomy cut is required; (ii) it ensures a more precise measurement of the wedge thickness, especially for wedges with considerable dimensions [8, 9]; (iii) this technique might be more familiar to the surgeon, who may use the surgical access to carry out associated procedures as well. The closing-wedge procedure can also overcome some of the disadvantages of LOW-DFO; for instance, the opening procedure requires bone grafting to fill and stabilize the osteotomy site in order to prevent excessive diastasis of bone fragments, with a delay in consolidation as a consequence. Fracture healing usually takes more time in LOW-DFO than in MCW-DFO, and LOW-DFO does not allow partial weight bearing immediately after surgery [10]. However, the X-ray-monitored angular correction and functional results are equivalent for the two techniques in the medium to long term [11], as is the conversion rate to knee prosthesis [12]. For these reasons, neither technique can be considered better than the other, but a thorough assessment of patient characteristics needs to be done. Therefore, in subjects who might be affected by bone healing defects, such as smokers or individuals with low bone quality, MCW-DFO should be preferred [13]. On the other hand, in patients who previously underwent lateral meniscectomy, the LOW-DFO technique should be preferred for its ability to compensate for subsequent substance loss. The MCW-DFO technique was chosen by the surgeon for the cohort of patients considered in the present work, as it was considered more appropriate to correct the actual site of deformity in these patients, meaning that an etiological treatment was needed instead of just a compensatory treatment. The aim of the present study was to retrospectively evaluate subjective radiological and clinical outcomes of medial closure supracondylar femoral osteotomies for arthritic valgus knee treatment at long-term follow-up.

## Materials and methods

All patients with a diagnosis of arthritic valgus knee who underwent medial closing-wedge supracondylar femoral osteotomy and were admitted to our department between 1 January 1980 and 31 December 2018 were included in the study; procedures were carried out by the same senior surgeon (M.B.), who used the same surgical technique consisting of a medial femoral osteotomy in minus with the placement of a plate with screws.

The following inclusion criteria were adopted for each patient: an available preoperative weight-bearing and anteroposterior full-length knee standing X-ray; specific surgery indications—lateral osteoarthritis (< Kellgren–Lawrence type II–III) with valgus alignment of the limb due to a valgus deformity of the distal femur, age < 55 years at the time of surgery, and BMI < 28; presence of the patient’s medical record in the archive. During preliminary record analysis, the following exclusion criteria were applied: patients who had been previously subjected to normocorrection osteotomy on the same limb; patients affected by inflammatory arthropathies or bone neoplasia; the occurrence of intra-articular fractures of the knee; ipsilateral hip prosthesis; extension of osteoarthritis to the medial or patellofemoral compartment.

Five knees (25%) had previously been treated with partial medial meniscectomy, associated in one case (5%) with a partial lateral meniscectomy. In three cases (15%), the anterior cruciate ligament (ACL) was reconstructed. A diagnostic arthroscopy was carried out during surgery in three cases (15%), and a partial lateral meniscectomy was performed in one of those cases.

Before surgery, the average BMI was 25.5 ± 3 (min. 20.3; max. 36.6), while it was 26.4 ± 2.5 (min. 21.5; max 31) at the final follow-up. The removed bone wedge had an average base thickness of 7 ± 2 mm and a measured opening angle of 9° ± 2°. At the final follow-up, a questionnaire was administered and an objective examination and a full-length knee standing X-ray were carried out.

Subjective parameters were evaluated through the subjective International Knee Documentation Committee (IKDC) scale, the Knee Society score (KSS) functional scale, the knee Injury and osteoarthritis outcomes score (KOOS), the Tegner activity scale, the visual analog scale (VAS), and the numeric rating scale (NRS-11). Objective parameters were analyzed using the objective KSS scale. In addition, the osteoarthritis grade was evaluated based on weight-bearing A-P X-rays and by using the Kellgren–Lawrence scale, and the axial deviation of the limb was gauged based on the full-length knee standing X-ray and by applying the hip–knee–ankle scale.

**Fig. 1:**
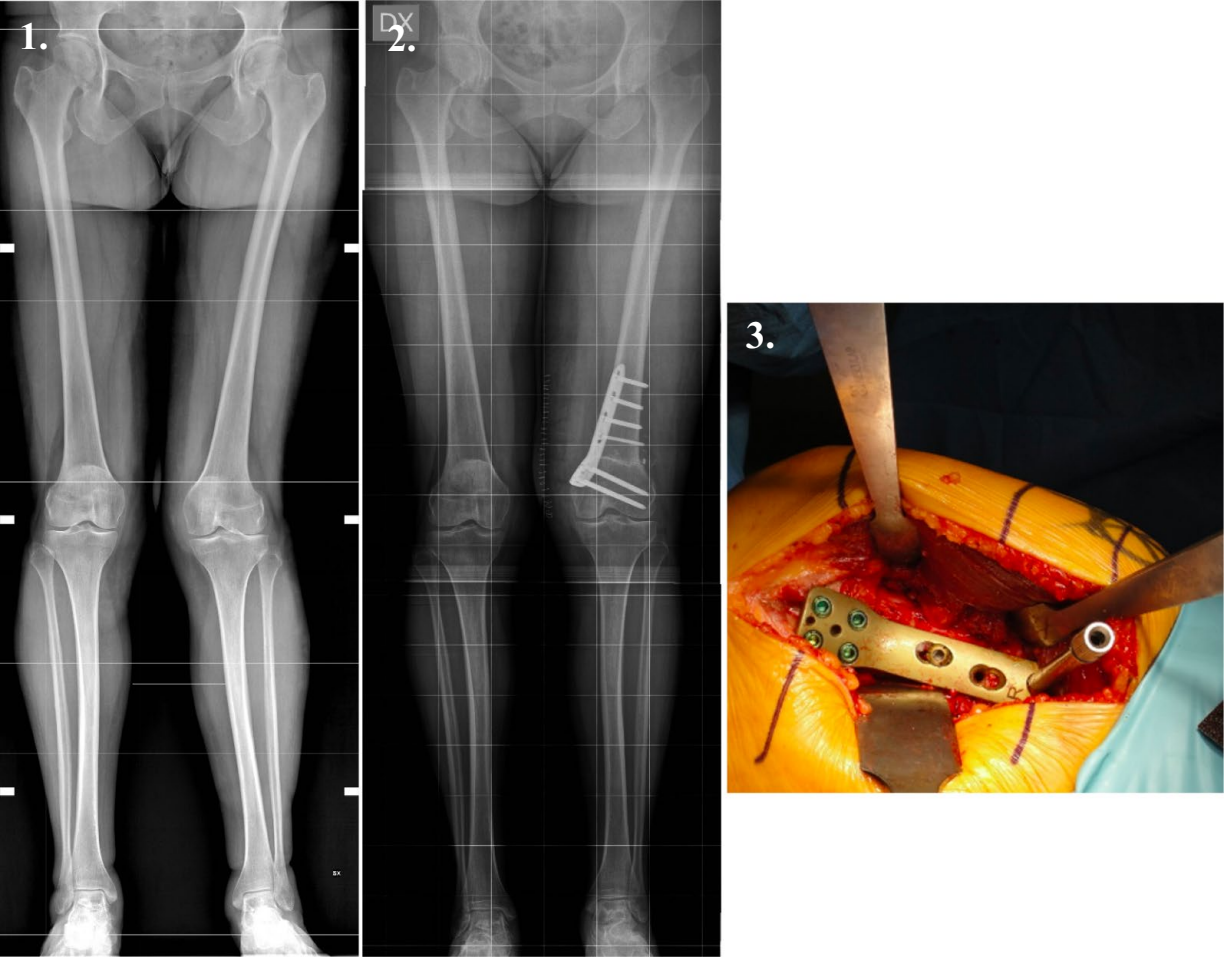
Image* 1* shows preoperative valgus lower limb alignment before femoral osteotomy; image* 2* shows lower limb alignment after correction using a medial distal femoral plate. Image* 3* shows an eight-hole Tomofix (DePuy Synthes) medial femoral condyle plate placed on the medial aspect of the femur, just after the reduction of the osteotomy line

Possible postoperative complications were reported, as well as associated procedures and previous or subsequent interventions. Data on the types of synthesis materials adopted were also collected: a Tomofix plate (DePuy Synthes, Raynham, MA, USA) was implanted in 9 cases (45%), while an AO 90° condylar plate was implanted in 11 patients (55%) (Fig. 1).

All patients involved signed an informed consent, in compliance with the protocol used by the local ethical committee.

### Surgical technique

The patient is placed in the supine position with hip and knee semiflexed at 50–60°, and a tourniquet is usually used. A medial-side distal femoral subvastus approach is used for this technique: a 10-cm skin incision is made starting from the medial femoral epicondyle and extending proximally along the shaft. The fascia is identified and is opened at the border of the vastus medialis muscle. The muscle is retracted anteriorly with two Hohmann retractors on the anterior femoral shaft and posteriorly with a blunt Hohmann to protect neurovascular structures. The periosteum is cut and, after checking the plate position under fluoroscopy, a double 2.0-mm Kirschner wire is passed from medial to lateral, parallel to the articular joint line, to guide the direction of the saw. The first osteotomy is performed following k-wires, making sure that the lateral femoral hinge is preserved. After calculating the height of the wedge (on preoperative planning), a second convergent osteotomy is performed. The bone wedge is removed and the osteotomy is completed by chisel. Finally, the osteotomy line is compressed by manual reduction. When the desired correction is achieved, the plate and screws are applied, starting from the distal part of the femur. After fluoroscopic examination, the wound is closed in layers and a compressive dressing is applied.

No brace is needed during the postoperative rehab program, which involves partial weight bearing (20 kg) with crutches for 45 days, exercises to promote quadriceps muscle recovery, and fully bending and extending the knee to gain its complete range of motion. After 45 days and an X-ray, the patient starts to recover full weight bearing.

### Statistical analysis

The Kolmogorov–Smirnov test was used to check that continuous variables were normally distributed. Statistical analysis was performed by comparing the preoperative data to the data obtained at the longest follow-up using a *t*-test. The following parameters were analyzed in this manner: subjective IKDC, subjective KSS, KOOS, Tegner, VAS, NRS-11, objective KSS, Kellgren–Lawrence scale, HKA index. In all statistical tests, a *p* value of < 0.05 was considered to indicate significance.

Survival rate evaluation was carried out using Kaplan–Meier curves.

## Results

**Fig. 2:**
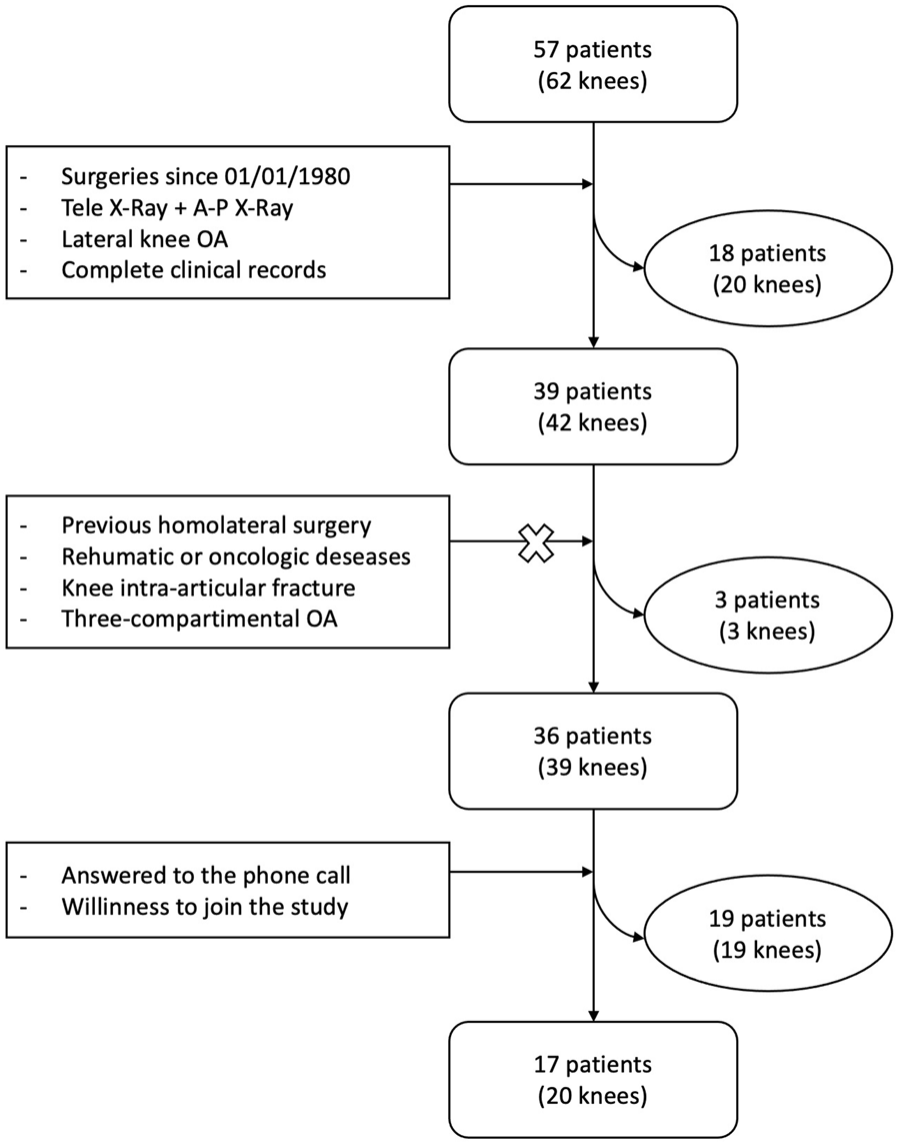
Flow chart of patient selection, from the initial group to the final subjects analyzed. Inclusion and exclusion criteria are shown in* square boxes*. The patients eliminated in each step are shown in the* oval boxes*

A total of 57 patients were enrolled, with five of those treated bilaterally (62 knees). Figure 2 shows the patient recruitment procedure step by step.

The mean follow-up at which patients were studied and re-evaluated was 138 ± 58 months (min. 63; max. 259), corresponding to 11 years and 6 months ± 4 years and 9 months (min. 5 years and 3 months; max. 21 years and 7 months).

The final sample included 17 patients (20 knees), three of whom were treated bilaterally. Twelve were female (70.6%) and 5 were male (29.4%). Mean age at intervention was 48 ± 9.8 years (min. 28; max. 61); 6 subjects (35%) were smokers while 11 (65%) were nonsmokers. Surgery was performed on 11 right knees (55%) and 9 left knees (45%).

Scores for subjective assessments of knee functionality and pain are shown in Table 1.

**Table 1:** Improvements in knee clinical scores and objective items from the preoperative period to the last examination

Scale	Preoperative	Final follow-up	*p* value
Subjective IKDC	39.4 ± 10.9	54 ± 15.2	< 0.01
Functional KSS	37.7 ± 10	63.9 ± 15.4	< 0.01
KOOS	45.7 ± 9.6	76.5 ± 10.5	< 0.01
Tegner	2.5 ± 0.7	3.1 ± 1.3	= 16
VAS	56.7 ± 12.9	42 ± 17.1	< 0.05
NRS-11	5.8 ± 1.1	4.4 ± 1.7	< 0.05
Objective KSS	42.2 ± 11.7	75 ± 22.5	< 0.01
Kellgren–Lawrence	II: 8 pts; III: 12 pts	II: 5 pts; III: 8 pts; IV: 7 pts	
Axial deviation (HKA)	8.6° ± 2°	1.3° ± 1.6°	< 0.01

Objective functionality as measured by the objective KSS scale was 42.2 ± 11.7 points preoperatively and 75 ± 22.5 during the last follow-up.

Osteoarthritis degree was evaluated using the Kellgren–Lawrence scale. Prior to the surgery, it was II in 8 cases and III in 12 cases; during the last follow-up, it was II in 5, III in 8, and IV in 7 cases. KL-scale grades in patients with a follow-up of > 4 years and < 10 years did not show any significant variation (*p* = 0.07) from a median value of II (range II–III) or 3 (range II–III); on the other hand, the KL-scale grades of patients with a follow-up of > 10 years increased significantly (*p* < 0.05) from a median value of II (range II–III) to III (range II–IV).

The axial deviation of the limb calculated via the HKA index was substantially modified, decreasing from an average value of 8.6° ± 2° preoperatively to 1.3° ± 1.6° at last follow-up (*p* < 0.01).

All parameters analyzed during follow-up showed an improvement compared to preoperative values.

Among the functionality indices, the subjective IKDC increased from a value of 39.4 ± 10.9 to 54 ± 15.2 (*p* < 0.01), the functional KSS increased from 42.2 ± 11.7 to 75 ± 22.5 (*p* < 0.01), and KOOS increased from 45.7 ± 9.6 to 76.5 ± 10.5 (*p* < 0.01).

The Tegner Activity Scale score increased from 2.5 ± 0.7 to 3.15 ± 1.3 (*p* = 0.14). The VAS score decreased from 56.75 ± 12.9 to 42 ± 17.1 (*p* < 0.05) and the NRS-11 score decreased from 5.8 ± 1.1 to 4.4 ± 1.7 (*p* < 0.05).

Objective functionality evaluated via the objective KSS showed an improvement from 39.2 ± 12.1 preoperatively to 63.8 ± 20.5 at last follow-up (*p* < 0.01).

Plate removal was required in 15 cases (75%) at a mean time of 18 ± 3 months.

A total knee arthroplasty (TKA) was required in 4 cases (20%) at an average time of 177 ± 56 months (min. 139; max. 259), corresponding to 14 years and 9 months (min. 11 years and 7 months; max. 21 years and 7 months).

At the final follow-up, 4 patients (20%) had been converted to total knee replacement.

**Fig. 3:**
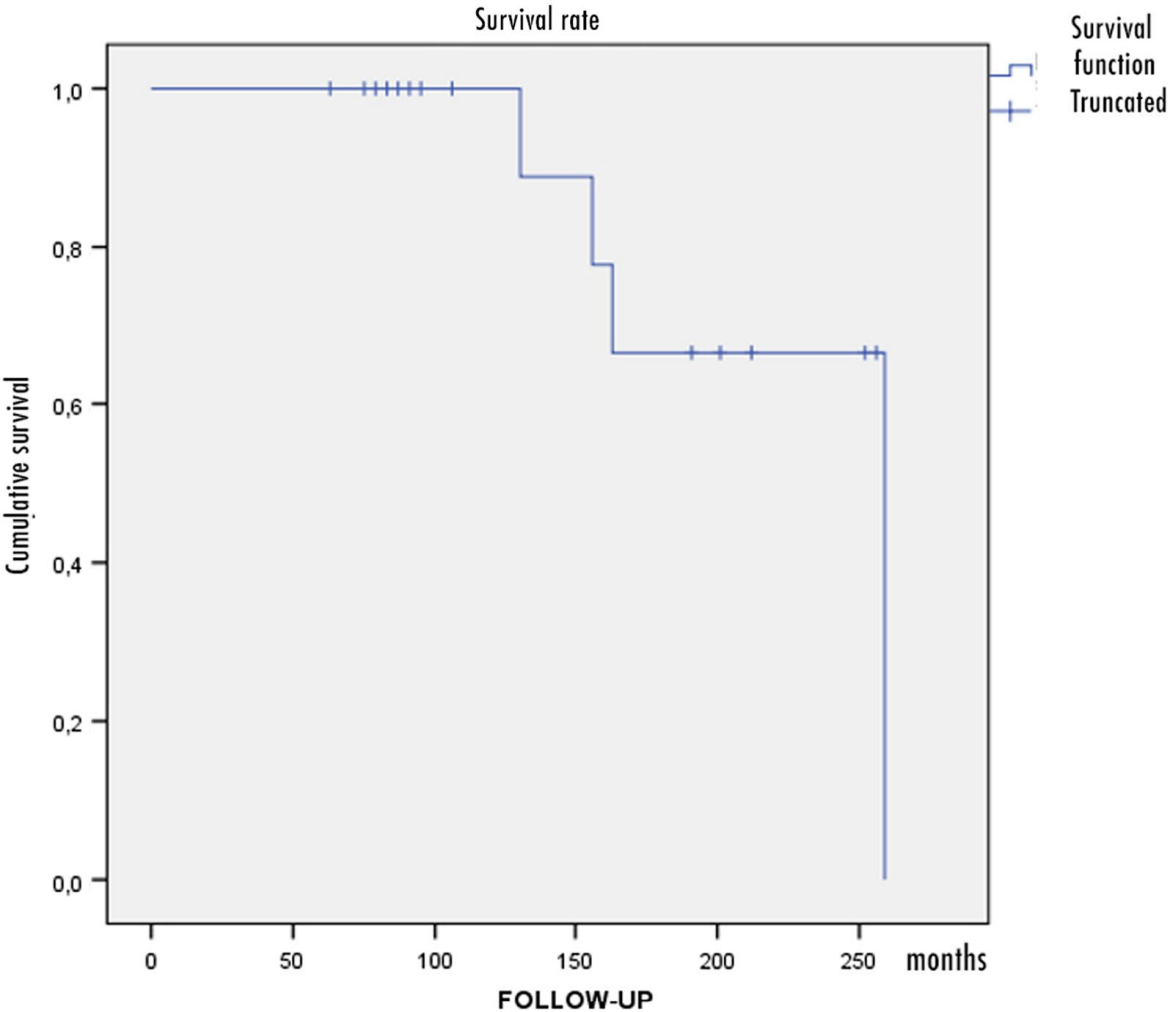
Kaplan–Meier survival rate curve showing conversion to TKA during the follow-up period. Survival was 100% (95% CI [100%, 93.1%]; *p* < 0.01) at a follow-up of 10 years and 66.7% (95% CI [74.1%, 59.3%]; *p* < 0.01) at a follow-up of 15 years

The estimated Kaplan–Meier curve survival rate was 100% (95% CI [100%, 93.1%]; *p* < 0.01) at 10 years (patients at risk: 17) and 66.7% (95% CI [74.1%, 59.3%]; *p* < 0.01) at 15 years (patients at risk: 13) (Fig. 3).

## Discussion

Our study confirmed that MCW-DFO is still an effective technique to treat valgus deviation in an arthritic knee. The study showed that it is a valid surgical option, especially in middle-aged patients. Therefore, it is important to evaluate the medium- to long-term results, including failures.

The average follow-up at which the 17 patients (20 knees) included in the study were re-evaluated was 11 years and 6 months (min. 5 years and 3 months; max. 21 years and 7 months). This is one of the longest reported in the literature, together with those in the studies by Forkel et al. [14] (mean follow-up 13.7 years; all KOOS scores increased significantly; no conversion to TKA; dropout rate 4%), Kosashvili et al. [15] (mean follow-up 15.1 years; survival at more than 15 years was 51.5%; dropout rate 8.3%; mean modified KSS score improved significantly (*p* < 0.01) from 36.8 preoperatively to 77.5 at 1 year), and Sternheim et al. [16] (mean follow-up 13.3 years; survival at 10, 15, and 20 years was 90, 79, and 21.5%, respectively; mean modified KSS score was 36.1 preoperatively, 74.4 at 1 year postoperatively, and 60.5 at last follow-up).

The MCW-DFO technique was chosen by the surgeon instead of the HTO procedure, as the former was considered more appropriate for correcting the actual site of the deformity, as already observed in 1985 by Maquet [17]. The MCW-DFO technique was chosen instead of LOW-DFO according to a series of technical considerations based on scientific reasons. For example, direct contact between bone surfaces at the osteotomy site and consequently an intrinsically stable fracture reduction tends to create a pressure by itself, enhanced by the femoral curvature. Another advantage is the reduced time before physical activity can be resumed when MCW-DFO is performed instead of LOW-DFO, as MCW-DFO allows the possibility of partial weight bearing on the limb from the start.

Clinical and X-ray results reported in the literature are identical for closure and opening techniques; there is no evidence that one technique is absolutely more effective than the other [18].

Radiographically, our results showed a significant reduction in lower limb axial malalignment; the mechanical axis obtained in our study matches that obtained in the series observed by Healy et al. [19] (2° valgus on average).

To assess the progression of osteoarthritis through imaging techniques, the patients were subdivided into two groups (follow-up < 10 years and > 10 years), who were then evaluated by using the KL scale. Nonprogression of the OA grade was observed for the group of patients who were followed up for between 4 and 10 years, from a median value of II (range II–III) to a median value of III (range II–III, *p* = 0.07). In the group of patients who were followed up for over 10 years, the OA grade changed from a median of II (range II–III) to a median of III (range II–IV, *p* < 0.05). These data seem to support the idea that the MCW-DFO procedure is capable of slowing down OA advancement in the medium to long term, even though it does not prevent the arthritic degenerative process connected to aging from taking place. Unfortunately, the sample size is too limited to draw clear statistical conclusions.

Comparing our results with those from other studies in the literature, some similarities and differences can be highlighted. The results for the subjective IKDC and KOOS scores reported by Buda et al. [20] (mean subjective IKDC increased from 44.06 to 80.09; mean KOOS increased from 45.21 to 79.59) were similar to those obtained in the present paper. Also, pain perception as quantified by VAS and NRS-11 significantly decreased in our study, additionally confirming the results reported by Buda et al. (who noted a decrease in average NRS-11 score from 6.1 to 2.7). We can declare that MCW-DFO can lead to satisfactory subjective results for the treatment of valgus arthritic knee. Pain reduction has a double effect on the quality of life since it both reduces discomfort and increases the opportunities to maintain a healthy level of physical activity and good functional ability. Although based on a larger sample size, Buda et al. reported that the Tegner activity scale score significantly increased (from 2.65 to 4.81 on average); in our study, such a significant change in the Tegner activity scale score was unlikely to be seen given that elderly patients have physiologically reduced activity levels that balance out the benefits of the surgery. Forkel et al. [14] also noted an increase in the Tegner activity scale score (from 3.5 to 4.2 on average), confirming the previously mentioned results. Comparing our results to those obtained by Sternheim et al. [16], we noted that the results of both studies overlapped when objective functionality was assessed through the KSS, with Sternheim et al. observing a significant improvement (from 36.5 to 63.1 on average), confirming that the results obtained in our study are supported by the current literature. Among the patients enrolled in our study, 4 cases (20%) underwent a TKA conversion. The survival rate at 10 years was 100%; at 15 years it reached 66.7%. The excellent results obtained in our study were more satisfactory than the results described for other studies: Backstein et al. [21], Finkelstein et al. [22], Sternheim et al. [16], and Wang and Hsu [23] obtained survival rates at 10 years ranging from a minimum of 64% to a maximum of 89.9%. Survival at 15 years was very low in the cases analyzed by Backstein et al. [21], reaching a value of 45%, while the highest value was 78.9%, as reported by Sternheim et al. [16].

In all cases subjected to TKA conversion, the indication for surgery was supported by a diagnosis of grade IV arthrosis based on the KL scale; unfortunately, the sample size was too limited to suggest a statistically significant relation between arthrosis grade and the need for a prosthetic replacement.

In our patient group, only one short-term complication was observed; it occurred in a patient diagnosed with paresthesia and allodynia affecting the anteromedial knee area, which was cured in 8 months. The main long-term complication was limited tolerance of synthesis materials, reported as a nuisance or light pain that led in 14 cases to the removal (70%) of the plate used for synthesis at an average follow-up of 19 ± 4 months; this removal rate is almost identical to that reported by Forkel et al. [14] (73% of cases).

Undoubtedly, our study has some limitations that are related to the retrospective approach used. Firstly, there was no control group due to the fact that is difficult to find patients with an arthritic valgus knee who were not subjected to any treatment. Secondly, only a limited number of patients were enrolled due to the low prevalence of the characteristics needed for DFO (patients who are young adults with a good level of physical activity, unicompartmental lateral osteoarthritis, and valgus deformity of the knee), despite the high number of patients admitted to our institute. Therefore, only 20 out of the 39 candidate knees were enrolled, leading to a 48.9% dropout rate, given that patients were either not found or not available to take part to the study.

Despite its limitations, the present study has clear clinical relevance and provides significant results that can be compared to case series already reported in the literature.

## Conclusions

In conclusion, results from the present study suggest that MCW-DFO can improve symptoms in patients with an arthritic valgus knee at medium- to long-term follow-up, reducing OA progression in carefully selected patients. This osteotomy can postpone the need for a prosthetic implant for over a decade, as the average TKA conversion time following MCW-DFO was 14 years.

## Data Availability

The datasets used and/or analyzed during the current study are available from the corresponding author on reasonable request.
